# Cost-effectiveness implications of GP intervention to promote physical activity: evidence from Perth, Australia

**DOI:** 10.1186/1478-7547-8-10

**Published:** 2010-05-13

**Authors:** Anura K Amarasinghe

**Affiliations:** 1 Centre for the Built Environment and Health, School of Population Health, The University of Western Australia, Australia

## Abstract

**Background:**

Physical inactivity is a major risk factor for many chronic diseases including diabetes, cardiovascular diseases and some cancers. It is estimated that, in Australia, physical inactivity contributes to 13,500 annual deaths and incurs an annual cost of AU$ 21 billion to the health care system. The cost of physical inactivity to the Western Australian (WA) economy is estimated to be about AU$ 2.1 billion. Increased burden of physical inactivity has motivated health professionals to seek cost effective intervention to promote physical activity. One such strategy is encouraging general practitioners (GPs) to advocate physical activity to the patients who are at high risk of developing chronic diseases associated with physical inactivity. This study intends to investigate the cost-effectiveness of a subsidy program for GP advice to promote physical activity.

**Methodology:**

The percentage of population that could potentially move from insufficiently active to sufficiently active, on GP advice was drawn from the Western Australian (WA) Premier's Physical Activity Taskforce (PATF) survey in 2006. Population impact fractions (*PIF*) for diseases attributable to physical inactivity together with disability adjusted life years (*DALYs*) and health care expenditure were used to estimate the net cost of intervention for varying subsidies. Cost-effectiveness of subsidy programs were evaluated in terms of cost per *DALY *saved at different compliance rates.

**Results:**

With a 50% adherence to GP advice, an annual health care cost of AU$ 24 million could be potentially saved to the WA economy. A *DALY *can be saved at a cost of AU $ 11,000 with a AU$ 25 subsidy at a 50% compliance rate. Cost effectiveness of such a subsidy program decreases at higher subsidy and lower compliance rates.

**Conclusion:**

Implementing a subsidy for GP advice could potentially reduce the burden of physical inactivity. However, the cost-effectiveness of a subsidy program for GP advice depends on the percentage of population who comply with GP advice.

## Introduction

The World Health Organisation (WHO) identified physical inactivity as a major risk factor contributing to diseases such as ischemic heart disease, ischemic stroke, breast cancer, colon/rectum cancer and diabetes mellitus [1]. It was estimated that, in Australia, physical inactivity contributes to 13,500 annual deaths and incurs an annual cost of AU$ 21 billion to the health care system [2,3]. The cost of physical inactivity to the Western Australian (WA) economy was estimated to be about AU $ 2.1 billion [2]. Increasing physical activity could potentially save at least 6.6% of total burden of diseases and injury in Australia [3]. In the UK, physical inactivity is directly responsible for 3% of disability adjusted life years lost and £1.06 billion direct health care cost to the National Health Service [4]. About CA$ 2.1 billion, or 2.5% of total direct health care costs in Canada, were attributable to physical inactivity in 1999 [5]. It was found that a 10% reduction in the prevalence of physical inactivity in Canada has the potential to reduce direct health care expenditure by CA$1,550 million per year [5]. In addition, in 1995, physical inactivity caused approximately 21,000 premature lives lost in Canada.

Increased burden of physical inactivity around the world has motivated health professionals to seek cost effective intervention to promote physical activity. One such strategy is encouraging general practitioners (GPs) to address health needs of patients who are at risk of developing chronic diseases associated with physical inactivity. One proposed Australian GP intervention aimed to tackle the obesity crisis and prevent chronic illnesses revealed that an overweight Australian could pocket a AU$ 170 subsidy by signing up for weight loss programs [6]. It was also indicated that the AU$ 200 cost of a 12-week weight loss program is currently beyond the reach of many people who could benefit from it. The Australian General Practice Network also wants AU$ 40 million to be spent on a national program to teach good parenting techniques [6].

Although a few attempts have been made to investigate the cost-effectiveness of physical activity intervention in primary care settings, all of them have a major drawback: the use of different health outcomes to assess health benefits [7-11]. Thus, results are difficult to compare across studies and programs. This may hinder or delay the implementation of the policies that may help promote physical inactivity in general. On the other hand, assessing cost-effectiveness in terms of common health outcomes may be more relevant for health advocates in allocating the limited healthcare budget available for prudent policy interventions. In the recent past, few attempts have been made towards this end in a cost-utility framework.

The cost utility analysis of physical activity counselling in general practice in New Zealand shows that the cost per quality adjusted life years (*QALY*) gained over full life expectancy ranges from NZ$ 827 to NZ$ 37516 ($AU 680 to 31,000). This study suggests that it would be wise encouraging GPs to prescribe physical activity advice in primary care settings [12]. The Active Script Programme (ASP) in Victoria, Australia was designed to increase the number of general practitioners (GPs) who delivered appropriate, consistent, and effective physical activity advice to patients. ASP showed that, although the impact of the GP intervention was modest, the cost-effectiveness figures were impressive. A study showed that the program only cost AU$ 138 per patient to become sufficiently active to a level that gains health benefits and a *DALY *can be saved at a cost AU$ 3647 per year [13]. One of the limitations of this study is that the modelling framework is based on a hypothetical % of people who become active, rather than the actual impacts of intervention. However, this is the only known Australian cost-effectiveness study which investigates the impacts of GP intervention in terms of cost per *DALY *saved.

Setting-specific promotions (e.g., in doctors surgeries, in recreational settings, etc.) and individually-focussed physical activity promotions have also shown to have modest success [14]. This study investigates this proposition by evaluating the cost-effectiveness of a subsidy program for GP advice to promote physical activity in Western Australia. It uses the best available information of survey data to assess the cost effectiveness of GP intervention in terms of cost per *DALY *saved.

## Analytical methods

### Welfare Implications and comparative statistics of Subsidized GP visits

A graphical welfare analysis of the implications of subsidized GP visits and a comparative statics analysis of subsidy on the demand for GP visit are available as additional file 1.

### Data

Primary data for this study were drawn from the Premier's Physical Activity Taskforce (PATF) survey conducted in 2006. This survey (N = 3361) measured the levels and types of physical activity among Western Australian adults (age 18 years and over) during November and December 2006. A balanced random sample of both men and women from all age groups 18 years and over were selected from four geographical regions including metropolitan Perth, Kimberly/Pilbara, Midwest/Goldfields and the South West.

Physical activity was determined against the self reported total time spent on vigorous-intensity physical activity, moderate-intensity physical activity and walking during the week. A sufficient level of physical activity threshold was identified as 150 minutes of moderate-intensity physical activity over five or more sessions or 60 minutes of vigorous-intensity physical activity in a week. This was based on the general physical activity guidelines recommended by the public health advocates including the Australian Government Department of Health and Aging [15-17]. Accordingly, participants were grouped into two physical activity categories namely sufficiently active (SA) (i.e. meets 150 or more minutes of moderate intensity physical activity) and insufficiently active (IA) (i.e. less than 150 minutes/week).

The survey also inquired from the participants whether they had received physical activity or exercise advice during their last visit to the doctor or GP. This information about physical activity advice was statistically analysed to project the impact of subsidy for GP advice to promote physical activity.

### Prevalence of insufficient/sufficient level of physical activity upon GP advice

The effectiveness of GP advice, i.e. the probability of being sufficiently active (SA) and insufficiently active (IA) given the GP advice (GA), was quantified by using PATF data. The probability of a person being sufficiently active when given the GP advice was quantified as . Invoking *Bayes theorem *it can be shown that *P*(*SA∩GA*) = *P*(*GA*|*SA*)*P(*SA*) or *P*(*SA*|*GA*)*P(*SA*).

Similarly, the probability of a person insufficiently active upon GP advice (i.e. *P*(*IA*|*GA*)) was quantified. We hypothesized that the difference between *P*(*SA*|*GA*) and *P*(*IA*|*GA*) reflects the proportion of population that could potentially be moved from insufficiently active to sufficiently active, upon GP advice.

### Population impact fractions (*PIF*)

Next, *PIFs *for diseases where physical inactivity is a risk factor for the % population that could potentially be transferred from insufficiently active (IA) to sufficiently active (SA) were derived. The *PIF *for a specific disease *i*, (PIF_i_) which is associated with physical inactivity was defined as *PFI*_*i *_= *PA*_*j*_(*RR*_*j*_-1)/1 + *PA*_*j*_(*RR*_*j*_-1) [18], where *PA*_j_, reflects the % population that could be transferred from insufficiently active (IA) to sufficiently active (SA) stage upon GP advice. *RR*_j _is the corresponding relative risk for disease *i *attributable to the insufficient level of physical activity. The term (*RR*_j_-1) indicates the excess risk faced by an insufficiently active person relative to the sufficiently active category. The relative risks of five diseases attributable to physical activity, were obtained from the Burden of Disease and Injury study in Australia [19]. Population impact fractions (*PIF*) for diseases were used to assess the potential burden that can be avoided in terms of disability adjusted life years (*DALYs*) and health care expenditure saved. In line with previous findings, the burden avoided was also allowed to vary with different compliance rates (i.e., % of people who adhere to GP advice). Previous findings have indicated that people who comply with GP advice for physical activity in the short term was about 20% [13]. The estimated health care cost-offsets were used to derive the net cost of GP intervention for varying subsidies.

### Potential burden avoided

We estimated the potential burden that can be avoided for five major diseases linked to physical inactivity in the Western Australian population: Colon Cancer, Heart Disease (HD) Stroke (ST), Type II Diabetes and Depression (DEP). The prevalence-based direct costs for five diseases were obtained from the health system expenditure on disease injury in Australia, 2000-01 [20]. Information about direct health care costs was related to hospital, medical, pharmaceutical, allied health research, public health and other associated costs for each of the major diseases attributable to physical inactivity. *DALYs *attributable to the five diseases were obtained from the burden of disease and injury study in Australia in the year 2003 [3]. Thus, all cost figures were adjusted in terms of year 2003 prices.

### Cost of subsidy for GP intervention

In reference to recent trends, this study assumed that an Australian made 6 GP visits/year on average [21]. In this analysis, it was also assumed that the patient could claim a subsidy of AU$ 20 per GP visit to get physical activity advice. This subsidy was also allowed to vary in the sensitivity analysis. Finally, the cost-effectiveness of subsidy programs were evaluated in terms of cost per *DALY *saved at different compliance rates.

## Results

About 15% of survey respondents (N = 541) reported to have received physical activity advice during their last visit to general practitioner (GP). Having received the GP advice, about 40% of respondents remained to be insufficiently active in comparison to 60% of sufficiently active. Thus, upon GP advice, it was hypothesized that about 20% of the population could potentially be moved from an insufficiently active to a sufficiently active stage.

Estimated burden in terms of health loss (*DALYs*) and health care expenditure averted are given in the tables 1 and 2. *PIFs *imply that about 16% of stroke and 12% of colon cancer attributable to physical inactivity could potentially be saved by means of GP involvement in physical activity advice. The results also suggest that GP advice can save 6,000 *DALYs *annually for the WA population. In addition, annual health care costs of AU $ 53 million could also be saved by the WA community.

**Table 1:** *DALYs *attributable to Five Diseases where Physical Inactivity is a Risk Factor in Western Australia (WA) and reduction in the Burden of Disease following GP advice

	*** Total DALYs lost each year in WA***^**a**^	***PIF***^**b**^	*Potential Annual DALYs gained with GP intervention*
***Diseases***			
Colon Cancer	5,721	0.123	703
Ischaemic Heart Disease	23,700	0.091	2,155
Stroke	10,655	0.167	1,776
Type 2 Diabetes	11,957	0.057	677
Depression	17,250	0.057	976

Total	69,281		6,286

a: calculated from the burden of disease and injury study in Australia in the year 2003.

b; based on relative risks of physical inactivity obtained form Burden of Disease and Injury study in Australia 1999.

**Table 2:** Total Health Care Cost in WA and Potential Cost offsets from the GP intervention for Five Diseases where Physical Inactivity is risk factor

Diseases	***Health care cost WA***^**c **^***($ Million)***	*PIF*	*Potential Cost Offsets (Million $)*
Colon Cancer	45	0.123	6.0
Ischaemic Heart Disease	178	0.091	16.0
Stroke	109	0.167	18.0
Type 2 Diabetes	99	0.057	6.0
Depression	135	0.057	8.0

Total	566		53.0

c; costs expressed in terms 2003 prices were calculated from health system expenditure on disease injury in Australia, 2000-01.

However, administering a subsidy for six annual GP visits at a rate of AU$ 20 with full compliance to the GP advice would cost AU$ 48 million to the WA economy. This yields a net saving of AU$ 5 million to the WA economy. As the % population who adhere to GP advice decreases, the subsidy program becomes a cost strategy as opposed to a net saving strategy. Reduction of compliance rate reduces the potential benefits gained from the GP advice.

Table 3 illustrates the health loss, health expenditure averted and cost-effectiveness for different subsidy and compliance rates. At a 75% compliance rate GP advice would yield an annual net cost of AU$ 12 million to the WA community. Thus a *DALY *can be averted at a cost of AU $ 2,649. If the compliance rate is reduced to 25%, cost per *DALY *saved would rise to AU $ 63,000 with a AU$ 50 subsidy for six annul visits.

**Table 3:** Cost-effectiveness for varying subsidy and compliance rates

Compliance Rate	100%	75%	50%	25%	20%	10%
Health loss (DALYs) averted	6,286	4,844	3,322	1,710	1377	697
Health expenditure averted ($ Million)	53	35	24	13	10	5
Cost effectiveness ($/DALY) with 20$ subsidy	(810)^e^	2,649	7,162	20,747	27,546	61,558
Cost effectiveness ($/DALY) with 25$ subsidy	1,099	5,126	10,775	27,762	36,263	78,781
Cost effectiveness ($/DALY) with 50$ subsidy	10,644	17,511	28,835	62,840	79,848	164,896

(.)^e^, indicates a dominant strategy where benefits gained or the value of burden avoided exceeds cost of subsidy.

## Discussion

This analysis showed that GP advice could potentially reduce the burden of physical inactivity. However, the success of a subsidy program for GP advice depends on the fraction of the population that complies with GP advice. GP advice to promote physical activity would be a dominant strategy with 100% compliance rate for a subsidy of AU$ 20 per visit and an average of 6 visits year. A patient could gain AU $180/year lump monetary benefit by seeing the GP for physical activity advice. However, 100% compliance rate is a conservative assumption in reality. With a 50% adherence to GP advice, an annual health care cost of AU$ 24 million could potentially be saved to the WA economy. A *DALY *can be saved at a cost of AU $ 11,000 with a AU$ 25 subsidy at a 50% compliance rate. Cost effectiveness of such a subsidy program decreases at higher subsidy and lower compliance rates. If higher compliance rates can be achieved, an even higher subsidy rate would be worth considering.

A previous study from Victoria, Australia found that GP intervention to promote physical activity can avert a *DALY *at a cost of AU$ 3,650 with a 20% short term compliance rate and a cost of AU$ 25 per consultation [13]. If the compliance rate were reduced to 5%, then the cost per *DALY *would rise to AU$ 9248. This study also has shown a similar trend with a lower compliance rate. However, the cost-effectiveness is slightly higher than the findings of the current study. A recent review of health promotion indicated that the median cost-effectiveness ratio of all health interventions in Australia was about AU$ 18,000 per *DALYs *averted or *QALYs *gained [22]. My results indicated that any subsidy of AU$ 20 or more for a GP visit with a 25% compliance rate would be above the Australian median cost-effectiveness standards. Previous studies however have claimed that AU$ 30,000 per *DALY *saved would be a favourable intervention in the Australian context [13]. According to WHO guidelines (i.e. less than three times GDP per capita for *DALY *averted), even a subsidy of AU$ 50 per GP visit with a 25% compliance rate would be justifiable [1].

It is quite clear therefore that the success of a subsidy for GP advice depends on the compliance rate (i.e. % of patients who adhere to GP advice and maintain a sufficient level of physical activity). Previous studies have emphasized that setting specific tailored interventions require multi-sectoral approaches beyond the general practitioner (GP) [13]. Tailored interventions should therefore focus on identifying physical, social, and psychological environments that may help improve health outcomes [23-25].

Many recent studies suggest that environmental interventions that give access to parks with scenic environments, multiple destinations, and sidewalks have the potential to increase physical activity and especially walking [26-28]. These complementary structures (for e.g., adequate sidewalks and parks for recreational walking) should be in place for GP advice to be effective. GP advice may not be a perfect substitute for other intervening strategies (for e.g., environmental intervention) or vice-versa to promote physical activity and allied health problems. Thus, policy makers should make prudent judgement of their willingness to trade-off buying health from different interventions. At an optimum, marginal health gains for the last dollar spent should be equal for all interventions although, in the presence of a wide range of epidemiological, medical, political and socio-economic disparities, setting priorities for public spending could be difficult.

However, it has been determined that GPs can play a key role in changing the behaviour of agents, as they were preferred and credible sources of health advice for the community [29]. GP involvement needs a concerted effort beyond clinical settings to raise community awareness by endorsing and recommending local programs, events and community participations that enhances physical activity. In doing so, GPs could implement a "five A's" model of prevention in which GPs assess, advise, agree, assist and arrange the patient's physical activity requirements [29]. This five "A" approach may lead to win-win welfare gains to the society as a whole.

It is also important to note that this study has several limitations. First, the primary reason for a GP visit has not been reported in the survey. It was assumed that participants who received advice visited the GP primarily because of a health problem related to physical inactivity. Second, the time passed since the last visit was not recorded in the survey. It was assumed that last visits to GP were made within one year of the time of the survey and on average a participant made 6 visits per year. Third, neither the information on participants who remained active upon GP advice, nor the subsequent quality of life has been reported. Fourth, the cost of intervention was based on a hypothetical subsidy program parallel to Medicare reimbursements. Fifth, this study relied on prevalence-based measures of costs and burden of disease rather than incidence-based measures which are potentially better for measuring the impact of a preventive policy. Finally, the unit disease costs and DALY's used related to two different time periods. However, potential underestimation of disease costs averted was minimized through the relevant price adjustment in comparison to DALY estimates. Despite these limitations, the projections made in this study using survey data may provide useful information to allocate limited health resources for cost-effective intervention to promote physical activity.

## Conclusion

This paper investigates the cost effectiveness of a subsidy program for general practitioner to promote physical activity in general populations. Results reveal that the subsidy for GP involvement to promote physical activity is cost effective, though the efficacy depends on compliance rates. A higher subsidy rate would be worth recommending if higher compliance rates could be achieved. Findings may be helpful in allocating healthcare resources for cost-effective intervention strategies in order to promote physical activity and public health.

## Competing interests

The authors declare that they have no competing interests.

## Authors' contributions

The author planned and designed the study. The views, opinions and conclusions expressed in this article are solely the responsibility of the author and do not necessarily represent the official view of the institute.

## Supplementary Material

Additional file 1 1. Rationale of a subsidy program for GP intervention; 2. Comparative statics of a subsidy on the demand for GP visits. Click here for file
