# Supplementary material for: Cost-effectiveness implications of GP intervention to promote physical activity: evidence from Perth, Australia
Source: Cost Eff Resour Alloc. 2010 May 13;8:10. doi: 10.1186/1478-7547-8-10 (PMC2887414; doi:10.1186/1478-7547-8-10)
Supplement: Additional file 1 — 1. Rationale of a subsidy program for GP intervention; 2. Comparative statics of a subsidy on the demand for GP visits. [file 1478-7547-8-10-S1.DOC]

# Additional Material:

**1. Rationale of a subsidy program for GP intervention**

The figure below illustrates the possible welfare implication of subsidized GP visits for physical activity advice.

###

$ P

**S**

**D**

**a**

**S’** buyers’ perspective

**b**

**o**

**e**

**d**

**c**

**S**

**S’**

**D’**

Q: Number of visits/year

**Q2**

**Q1**

With the assumption of an ideal market situation, DD and SS represent the demand for and supply of GP services. From the buyers perspective, a subsidy would cause the supply curve to move form SS to SS’, where a patient would pay a lower price for GP advice at point **‘c’**. Similarly, with a subsidy, the GP gets a higher price at point **‘d’**. As a result of the subsidy, patient visits for GP advice will increase from Q1 to Q2. Thus, the total cost of a subsidy, **abcd**, is paid by the government. However, if the sum of welfare changes (i.e., consumer and producer surplus) **(aboe + eocd),** is less than the government expenditure, society would end up with a net welfare loss represented by the area of **obc.** The government supported subsidy program has then caused a net welfare loss to society**.** This underlies the rationale for cost effective interventions to promote physical activity.

**2. Comparative statics of a subsidy on the demand for GP visits**

Having analysed the welfare implications graphically, I next turn to the comparative statics of a subsidy policy on GP visits. Let the demand and supply functions for GP visits be

$Qd=D(P,Y)$ **(1)**

$Qs=S(P,T)$ **(2)**

where $Qd$ and $Qs$ are demand for and supply of GP services, P is the price of GP services, Y and T are the exogenous income of an individual and the subsidy for GP services. By total differentiation, it can be shown that

$dQ_{p}=\frac{\partial D(.)}{\partial P}*dP+\frac{\partial D(.)}{\partial Y}*dY$ **(1a)**

$dQ_{s}=\frac{\partial S(.)}{\partial P}*dP+\frac{\partial S(.)}{\partial T}*dT$ **(2a)**

The first order conditions;$\frac{\partial D(.)}{\partial p}=D_{P}<0$; and $\frac{\partial D(.)}{\partial Y}=D_{Y}>0$ are the incremental effect of price and income on the demand for GP visits . Similarly $\frac{\partial S(.)}{\partial P}=S_{P}>0$and $\frac{\partial S(.)}{\partial T}=S_{T}>0$ are incremental supply of GP services with respect to price and subsidy respectively. At equilibrium, where supply equals demand, the $dQ_{p}=dQ_{s}=dQ$. By rearranging **2a**, it can be shown that

$dQ=Dp*\left(\frac{dQ-S_{T}*dT}{S_{P}}\right)+Dy*dY$By further simplification, $dQ=\frac{Sp}{Sp-Dp}\left(Dy*dy-Dp*\frac{St*dT}{Sp}\right)$**.** If $dy=0$ then $dQ=\frac{Sp}{Sp-Dp}\left(-Dp*\frac{St*dT}{Sp}\right)$

Thus, the incremental effect of demand for GP advice with respect to a subsidy equals: $\frac{dQ}{dT}=\frac{-\mathop{Dp}\limits^{-} *\mathop{St}\limits^{+} }{\mathop{Sp}\limits_{+} -\mathop{Dp}\limits_{\_} }\succ0$**.** This implies that a subsidy can raise the number of GP visits for physical activity advice.
